# Meat bezoar-induced small bowel obstruction: a rare but critical cause of acute intestinal distress

**DOI:** 10.1093/jscr/rjaf448

**Published:** 2025-06-25

**Authors:** Yassine El Bouazizi, Amine El Bouazizi, Youssef Yaikoubi, Sabrillah Echiguer, Taha Kabbaj, Zakaria El Mouatassim, Oumaima Lahnaoui, Amine Benkabbou, Mohammed Anass Majbar, Amine Souadka

**Affiliations:** Surgical Oncology Department, National Institute of Oncology, Ibn Sina University Hospital, Rabat, Morocco; General Surgery Department, Ibn Sina University Hospital, Morocco; Private General Surgery Center, Morocco; Surgical Oncology Department, National Institute of Oncology, Ibn Sina University Hospital, Rabat, Morocco; Surgical Oncology Department, National Institute of Oncology, Ibn Sina University Hospital, Rabat, Morocco; Surgical Oncology Department, National Institute of Oncology, Ibn Sina University Hospital, Rabat, Morocco; Surgical Oncology Department, National Institute of Oncology, Ibn Sina University Hospital, Rabat, Morocco; Surgical Oncology Department, National Institute of Oncology, Ibn Sina University Hospital, Rabat, Morocco; Surgical Oncology Department, National Institute of Oncology, Ibn Sina University Hospital, Rabat, Morocco; Surgical Oncology Department, National Institute of Oncology, Ibn Sina University Hospital, Rabat, Morocco

**Keywords:** meat bezoar, small bowel obstruction (SBO), liver parenchyma, surgical management

## Abstract

Acute intestinal obstruction is a common surgical emergency, usually caused by adhesions, tumors, or hernias. Bezoars are a rare but significant cause of small bowel obstruction (SBO), even in patients without prior gastrointestinal surgery. We report a case of a 60-year-old man with no surgical history who presented with nausea, vomiting, abdominal pain, and absence of bowel movements. Imaging suggested SBO, and diagnostic laparoscopy confirmed a meat bezoar obstructing the terminal ileum. Histopathological examination revealed thermally altered liver parenchyma, consistent with undigested cooked meat. The bezoar was successfully removed surgically, and the patient recovered without complications. This case underscores the importance of considering bezoars, including rare forms like meat bezoars, in the differential diagnosis of SBO, particularly in patients with nonspecific symptoms. Early recognition and surgical intervention are vital to avoid serious complications such as perforation or ischemia.

## Introduction

Acute intestinal obstruction is a common surgical emergency, responsible for 15% of hospital admissions for acute abdominal pain [1], with ~80% involving the small intestine. Common causes include adhesions, tumors, hernias, and inflammatory diseases [2]. Bezoars, though rare, account for 0.4%–4% of mechanical obstructions, even in patients without prior gastric surgery [3].

Bezoars are classified into phytobezoars, trichobezoars, pharmacobezoars, and lactobezoars. Meat bezoars, among the rarest, form from undigested meat fibers caused by poor mastication or impaired motility. Risk factors include diabetes, hypothyroidism, and psychiatric conditions like trichophagia. Diagnosing meat bezoars preoperatively is challenging due to nonspecific symptoms [4, 5].

We report a case of meat bezoar-induced small bowel obstruction (SBO) in a patient with no surgical history.

## Case report

A 60-year-old man presented with nausea, vomiting, abdominal pain, and no bowel movement or flatus for 3 days. He was hemodynamically stable and afebrile. Abdominal exam revealed distension and mild tenderness. Bowel sounds were hyperactive.

Computed tomography (CT) scan showed small bowel dilatation and terminal ileum thickening, suggesting SBO and possible Crohn’s disease. Initial management included bowel rest, nasogastric decompression, and IV fluids. Symptoms persisted with fecaloid vomiting.

**Figure 1:**
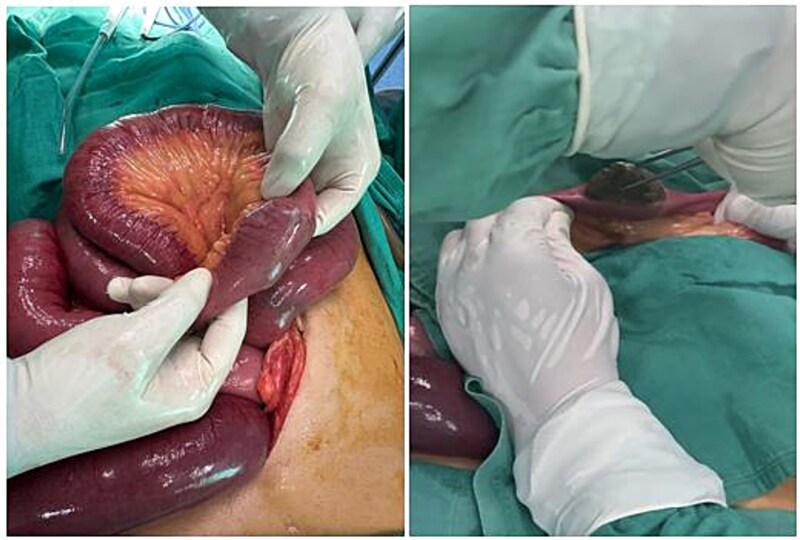
The meat bezoar is seen completely obstructing the small bowel lumen.

**Figure 2:**
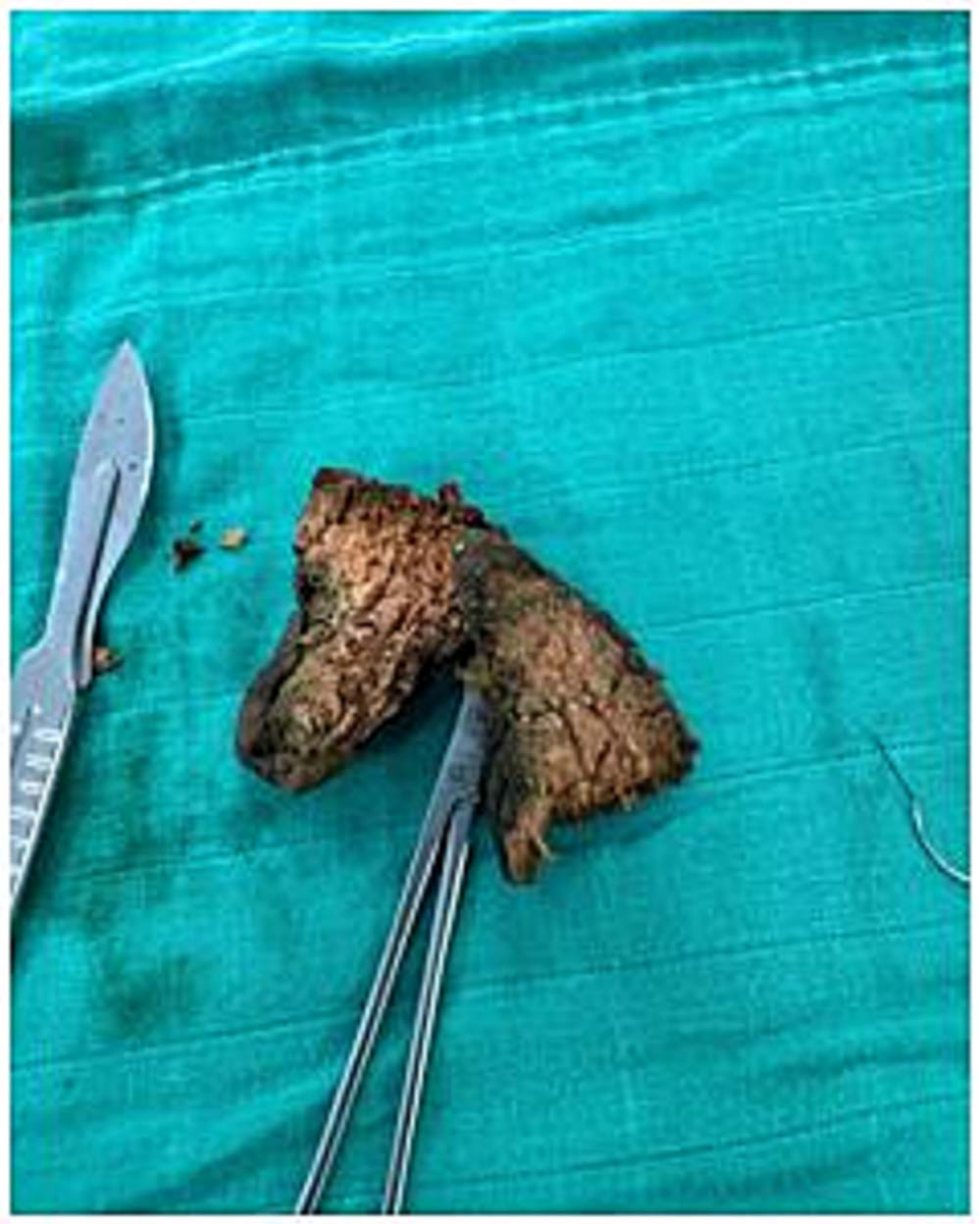
The meat bezoar, composed of cooked and undigested food (identified as liver parenchyma), is removed from inside of the small bowel.

**Figure 3:**
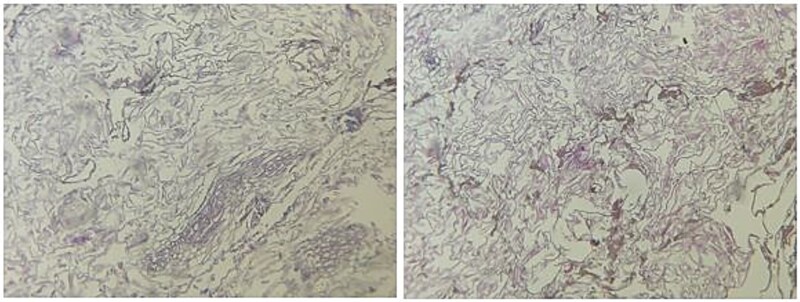
Histopathological alterations of liver parenchyma: microscopic study.

Laparoscopy revealed dilated small bowel and a mass in the terminal ileum. An enterotomy (Fig. 1) allowed removal of a 5 × 2 × 2.5 cm meat bezoar (Boulfafoma). Histopathology identified liver parenchyma fragments measuring 4.5 × 3 × 2.5 cm and 5 × 2 × 2.5 cm (Fig. 2). Microscopy showed dystrophic liver with liquefactive and ischemic necrosis and ghost-like lobules, consistent with cooked liver (Fig. 3).

Postoperative recovery was uneventful, and the patient was discharged with outpatient follow-up.

## Discussion

Meat bezoars, although rare, are a significant cause of small bowel obstruction (SBO), often resulting from inadequate mastication, which leads to the accumulation of large, indigestible food particles within the gastrointestinal tract. While bezoars are frequently associated with gastrointestinal surgery, especially gastrectomy or procedures that impair gastric motility and acid secretion, they can also form in patients without prior surgical interventions. This emphasizes the importance of considering bezoars as a potential cause of SBO in all patients, even those without a significant medical or surgical history [6].

The pathophysiology of meat bezoars involves the ingestion of large chunks of poorly masticated meat that resist normal enzymatic digestion in the stomach. These materials, unable to break down properly, move into the intestines, where they can cause obstruction. Bezoar formation is promoted by factors such as poor dental health, eating disorders, neurological conditions affecting motility (e.g. Parkinson’s disease), or psychiatric disorders that lead to compulsive eating behaviors like trichophagia (eating hair) or pica (eating non-food objects) [7]. In this case, the patient had no prior gastrointestinal surgery or significant medical conditions, making it an unusual presentation of a meat bezoar. It may be linked to dietary habits, particularly inadequate mastication of large pieces of meat.

The clinical presentation of a meat bezoar-induced SBO is often nonspecific, mimicking other abdominal distress causes. Typical symptoms include nausea, vomiting, abdominal pain, bloating, and cessation of bowel movements or gas. In our case, the patient’s initial symptoms were nonspecific, and imaging studies, including a CT scan, showed signs of SBO with dilated bowel loops and air-fluid levels, but no specific etiology was immediately apparent. The CT scan revealed thickening of the terminal ileum, suggesting Crohn’s disease or other causes of ileal inflammation. These findings were later clarified by diagnostic laparoscopy, which revealed a foreign body obstructing the terminal ileum, ultimately confirming the diagnosis of a meat bezoar [8, 9].

Histopathological examination of the excised bezoar revealed liver parenchyma with features of dystrophic changes, including liquefactive and ischemic necrosis, consistent with thermal alteration, as might occur with cooked liver. This suggested that the bezoar had formed from meat, specifically cooked liver, that was not sufficiently broken down before ingestion [10].

Meat bezoars are often initially misdiagnosed due to their nonspecific presentation. Delayed diagnosis can lead to serious complications such as bowel perforation, ischemia, and necrosis if left untreated. Therefore, it is critical to consider bezoars in the differential diagnosis of SBO, especially when common causes such as adhesions, tumors, or hernias are ruled out. In this case, despite initial conservative management, the patient’s symptoms persisted, and his condition deteriorated. The failure of measures such as bowel rest, nasogastric decompression, and intravenous fluids required surgical intervention to prevent further complications like bowel ischemia or perforation [11].

Surgical intervention, typically via laparoscopy, is the gold standard for diagnosing and managing bezoars that do not respond to conservative measures. Laparoscopy offers direct visualization of the obstructed bowel segment, facilitating the removal of the bezoar with minimal surgical trauma. In this case, where laparoscopy was unavailable, open surgery was performed to remove the bezoar from the terminal ileum. Fortunately, the patient’s condition improved immediately after surgery, and he remained asymptomatic postoperatively.

This case highlights several important points in managing bezoars, particularly meat bezoars. First, it underscores the importance of considering bezoars in the differential diagnosis of SBO, especially in patients with nonspecific symptoms. Second, it emphasizes the role of imaging, particularly CT scans, in detecting signs of bowel obstruction and aiding in the diagnosis of bezoars. Finally, it stresses the need for prompt surgical intervention when conservative management fails, as delaying treatment can lead to severe complications, including bowel perforation, sepsis, and long-term gastrointestinal dysfunction.

## Conclusion

Meat bezoars, although rare, should be considered in the differential diagnosis of acute small bowel obstruction after ruling out common causes like adhesions, tumors, and hernias. Early diagnosis and surgical intervention are vital to prevent life-threatening complications such as bowel perforation and ischemia. This case emphasizes the importance of considering bezoars in patients with nonspecific gastrointestinal symptoms, even without prior surgery, and highlights the value of imaging and laparoscopy in effective management. The patient’s favorable outcome demonstrates the benefits of timely recognition and treatment.
